# Oxymatrine induces anti-tumor response in cervical cancer by modulating circ_0008460/miR-197-3p/ribonucleotide reductase subunit M2 (RRM2)

**DOI:** 10.1080/21655979.2022.2078943

**Published:** 2022-05-24

**Authors:** Siwei Li, Heng Zhang, Yunping Jiao, Xiao Song, Lei Wei, Xing Liu

**Affiliations:** a Pharmacy Department, Northwest Women and Children Hospital, Xi’an, Shaanxi, China; b Clinical Pharmacy Department, the Second People’s Hospital of Shaanxi Province, Xi’an, Shaanxi, China; c Obstetrics Department, Northwest Women and Children Hospital, Xi’an, Shaanxi, China

**Keywords:** Circ_0008460, oxymatrine, cervical cancer, miR-197-3p, RRM2

## Abstract

Oxymatrine (OMT) has exhibited an anti-cancer role in human cancers, including cervical cancer (CC). The dysregulated circular RNAs (circRNAs) are key regulators in cancer biology, and circ_0008460 was upregulated in CC. This study was performed to investigate the circRNA-based molecular mechanism for OMT in CC. RNA detection for circ_0008460, microRNA-197-3p (miR-197-3p), or ribonucleotide reductase subunit M2 (RRM2) was completed using reverse transcription-quantitative polymerase chain reaction assay. Cell behaviors were assessed by Cell Counting Kit-8 assay for cell viability, colony formation assay or Edu assay for cell proliferation, flow cytometry for cell apoptosis, and wound healing assay/transwell assay for migration/invasion. Protein expression examination was conducted using western blot. Dual-luciferase reporter assay and RNA pull-down assay were applied to confirm target binding. Tumor xenograft assay was performed for OMT research *in vivo*. OMT induced circ_0008460 downregulation in CC cells. OMT-induced inhibitory effects on cell growth, migration, and invasion but promoting effect on cell apoptosis were attenuated by circ_0008460. Circ_0008460 directly interacted with miR-197-3p, and OMT inhibited malignant behaviors of CC cells via mediating circ_0008460/miR-197-3p axis. RRM2 acted as a target for miR-197-3p and circ_0008460 affected the RRM2 level through absorbing miR-197-3p. OMT upregulated miR-197-3p to inhibit RRM2 expression to impede CC cell development. CC tumorigenesis was suppressed by OMT via targeting circ_0008460/miR-197-3p/RRM2 axis *in vivo*. These results suggested that OMT restrained CC cell progression *in vitro* and tumor growth *in vivo* by downregulating circ_0008460 to mediate miR-197-3p/RRM2 axis.

## GRAPHICAL ABSTRACT

**Figure:**
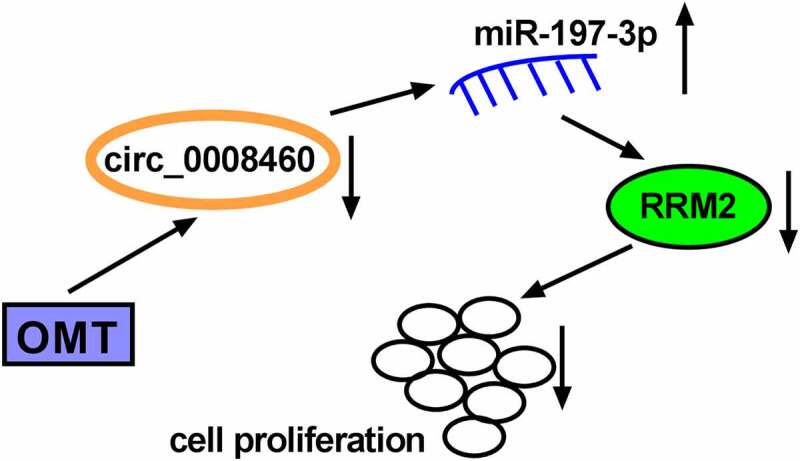

## Highlights

- Circ_0008460 reverses oxymatrine-induced inhibitory effects on cervical cancer cells.
- Circ_0008460 acts as a miR-197-3p sponge to affect RRM2 expression.
- Oxymatrine reduces tumor growth *in vivo* via inhibiting circ_0008460 to regulate miR-197-3p/RRM2 axis.

## Introduction

Cervical cancer (CC) is the leading cause of cancer-induced morbidity and mortality of women in low/middle-income countries globally [1]. Although treatment technology has been advanced for low-risk and early-stage diseases, the overall prognosis is still poor in CC patients with metastatic and recurrent diseases [2]. Oxymatrine (OMT) is an important active ingredient of Sophora roots, and it exhibits anti-cancer effects on malignant phenotypes of CC cells [3,4]. However, how OMT acts in CC progression remains unclear at the molecular level.

Circular RNAs (circRNAs) are specific products of back-splicing and have an aberrant expression in a wide variety of cancer types [5]. Increasing circRNAs are involved in various kinds of cellular processes in CC progression [6]. Circ_0000285 has been manifested to facilitate metastasis and colony formation of CC cells [7]. Also, circRNA plasmacytoma variant translocation 1 (circRNA_PVT1) promoted CC cell invasion and migration [8]. Circ_0008460 is derived from Wolf–Hirschhorn syndrome candidate gene-1 (WHSC1), and circRNA microarrays showed that circ_0008460 was abnormally overexpressed in CC cells [9]. The role of circ_0008460 in CC progression and its association with OMT need further exploration.

In addition, circRNAs are known to function as oncogenic or inhibitory regulators in CC by controlling microRNAs (miRNAs) to affect the expression levels of downstream mRNAs [10]. For example, circ_0000326 knockdown resulted in cell cycle retardation and proliferation inhibition by regulating miR-338-3p-targeted cyclin-dependent kinase 4 (CDK4) expression in CC cells [11]. Circ_0000515 was suggested to accelerate CC cell development by leading to upregulation of the ETS-domain-containing protein (ELK1) via sequestering miR-326 [12]. Gu *et al*. found that miR-197-3p impeded cell malignant behaviors in CC, and circRNA zinc finger protein 609 (circZNF609) regulated E2F transcription factor 6 (E2F6) level via sponging miR-197-3p [13]. Many miRNAs are involved in the regulation of CC progression via targeting genes. For instance, miR-873-5p promoted CC development via downregulating ZEB1 and miR-497-5p acted as an anti-cancer molecule by targeting Fatty Acid Synthase (FASN) in CC [14,15]. Ribonucleotide reductase subunit M2 (RRM2) was validated as an oncogene in CC, and miR-140-3p or miR-5095 directly targeted RRM2 to induce expression downregulation of RRM2 [16,17]. Whether circ_0008460 can sponge miR-197-3p to mediate RRM2 level has not been expounded.

The aim of this study was to explore the molecular mechanism for OMT in CC. Herein, circ_0008460 was hypothesized to regulate the expression of RRM2 through targeting miR-197-3p, thus participating in the biological role of OMT in the development of CC.

## Materials and methods

### CC and normal tissues

Sixty patients with CC were enrolled for the current research. CC tissues (*n* = 60) were collected during surgical excision at the Northwest Women and Children Hospital, and adjacent tissues (*n* = 60) were used as normal controls. There was no medical treatment before surgery of patients. All tissue samples were saved at −80°C for further extraction of RNA or protein. This research strictly followed the Declaration of Helsinki involving human subjects and it has been approved by the Ethics Committee of Northwest Women and Children Hospital.

### Cell culture and OMT treatment

CC cell lines (CaSki, SiHa) and normal control (Ect1/E6E7) were bought from BeNa Culture Collection (BNCC; Beijing, China). Cell culture with Dulbecco’s modified Eagle medium (Gibco, Carlsbad, CA, USA) was carried out in a 5% CO_2_, 37°C incubator. Ten percent fetal bovine serum (FBS; Gibco) and 1% antibiotic solution (Gibco) were added into basic medium to maintain cell growth. In addition, cell medium was added with mycoplasma inhibitors to prevent mycoplasma infection. All cell lines were identified to be without pollution. CaSki and SiHa cells were exposed to OMT (Selleck, Houston, TX, USA) with 2 mg/mL, 4 mg/mL, or 6 mg/mL.

### Cell transfection

The pcD5-ciR vector (vector; GENESEED, Guangzhou, China) was cloned with circ_0008460 sequence to generate overexpression vector pcD5-ciR-circ_0008460 (oe-circ_0008460). Mimic and inhibitor for miR-197-3p (miR-197-3p/anti-miR-197-3p), mimic and inhibitor controls (miR-NC/anti-miR-NC), small interfering RNA targeting circ_0008460, and negative control (si-circ_0008460, si-NC) were acquired from RIBOBIO (Guangzhou, China) for expression upregulation or knockdown. In addition, the lentiviral overexpression vector of circ_0008460 (lenti-circ_0008460) and control group (lenti-NC) were used for stable overexpression *in vivo* assay. A total of 1 × 10^5^ CaSki and SiHa cells were seeded into 96-well plates and cultured to 70% coverage; then, vectors and RNAs were transfected with the Lipofectamine™ 3000 Kit (Invitrogen, Carlsbad, CA, USA).

### Reverse transcription-quantitative polymerase chain reaction (RT-qPCR) assay

Trizol reagent (Solarbio, Beijing, China) was applied for acquisition of total RNA, followed by reverse transcription and PCR preparation using RevertAid First Strand cDNA Synthesis Kit and TaqMan One-Step RT-qPCR Kit (Solarbio). Primer sequences are exhibited in Table 1. The PCR system was amplified on ABI 7500 Real-Time PCR system (Applied Biosystems, Foster City, CA, USA). The relative expression levels were analyzed via the 2^−∆∆Ct^ method [18], with glyceraldehyde-phosphate dehydrogenase (GAPDH) and U6 as endogenous reference genes. Additionally, total RNA was added with 5 U/μg RNase R (GENESEED) and incubated at 37°C for 1 h. Then the stability of circ_0008460 was assessed via RT-qPCR.

**Table 1.** Primer sequences for RT-qPCR

Name	Primers for PCR (5’-3’)	
hsa_circ_0008460	Forward	TGGTGTGGTCCAAAGTGTCG
	Reverse	ATTCCATCCAGCCCAGATGC
WHSC1	Forward	CGGAAGAGGGAGACAAGCAA
	Reverse	AGCCCGATTTCGCTTCTTCA
miR-136-5p	Forward	GTATGAACTCCATTTGTTTTGAT
	Reverse	CTCAACTGGTGTCGTGGAG
miR-142-3p	Forward	GTATGATGTAGTGTTTCCTACTT
	Reverse	CTCAACTGGTGTCGTGGAG
miR-197-3p	Forward	GTATGATTCACCACCTTCTCCA
	Reverse	CTCAACTGGTGTCGTGGAG
miR-335-5p	Forward	GTATGATCAAGAGCAATAACGAA
	Reverse	CTCAACTGGTGTCGTGGAG
miR-421	Forward	TCGGCAGGATCAACAGACATTAATT
	Reverse	CTCAACTGGTGTCGTGGAG
miR-516b-5p	Forward	TCGGCAGGATCTGGAGGTAAGAAG
	Reverse	CTCAACTGGTGTCGTGGAG
miR-579-5p	Forward	TCGGCAGGTCGCGGTTTGTGCCAG
	Reverse	CTCAACTGGTGTCGTGGAG
miR-888-5p	Forward	TCGGCAGGTACTCAAAAAGCTGT
	Reverse	CTCAACTGGTGTCGTGGAG
RRM2	Forward	GCGCGGGAGATTTAAAGGC
	Reverse	TCCTTGTCGACCAAGCTGAG
GAPDH	Forward	GACAGTCAGCCGCATCTTCT
	Reverse	GCGCCCAATACGACCAAATC
U6	Forward	CTCGCTTCGGCAGCACA
	Reverse	AACGCTTCACGAATTTGCGT

### Cell Counting Kit-8 (CCK-8) assay

Cell viability detection was implemented using the CCK-8 assay. A total of 1 × 10^5^ cells/well were seeded into the 96-well plates overnight, and cells were incubated with 10 μL/well CCK-8 solution (Solarbio) for 4 h; then, optical density values at 450 nm in CaSki and SiHa cells were read by a microplate reader (Bio-Rad, Hercules, CA, USA).

### Colony formation assay

CaSki and SiHa cells were transplanted into 12-well plates with 500 cells/well and then cultured in a 37°C incubator. After two weeks, white colonies were observed and stained with 0.1% crystal violet (Beyotime, Shanghai, China) for 10 min. Then, stained colonies were counted via Image J software (NIH, Bethesda, MD, USA).

### Edu assay

Edu cell proliferation Kit (Beyotime) was used for the determination of proliferation [19]. Briefly, 5 × 10^4^ CaSki and SiHa cells were incubated with 100 µL Edu solution and 100 µL diamidine phenylindole (DAPI; Solarbio) according to the instruction book. Cells were observed under a fluorescence microscope (Olympus, Tokyo, Japan); then, Edu and DAPI merged cells were counted as Edu-positive cells.

### Flow cytometry

Cell apoptosis was assessed using flow cytometry as previously described [20]. Annexin V Apoptosis Kit (BD Biosciences, San Diego, CA, USA) was employed to examine apoptotic cells. A total of 1 × 10^5^ CaSki and SiHa cells were collected into new tubes at 72 h post-transfection and then pipetted with 10 µL Annexin V-fluorescein isothiocyanate (Annexin V-FITC) and 5 µL propidium iodide (PI). Twenty minutes later, cells were analyzed through a flow cytometer (BD Biosciences). FITC^+^/PI^−^ or FITC^+^/PI^+^ cells were defined as viable apoptotic cells and non-viable apoptotic cells, respectively.

### Western blot

Total protein isolation was performed by Radioimmunoprecipitation assay buffer and concentration detection was conducted by BCA Protein Assay Kit, according to the manufacturer’s guidelines. Then, protein blots were examined as previously reported [21,22]. Protein bands were visualized by Electrochemiluminescence (ECL) Ultra Western HRP Substrate, and level analysis was performed using Image J software (NIH). The reagents were commercially provided by Sigma-Aldrich (St. Louis, MO, USA). The information of primary antibody is as follows: B-cell lymphoma-2 (Bcl-2; Abcam, Cambridge, UK, ab32124, 1:1000), Bcl-2-associated X (Bax; Abcam, ab32503, 1:1000), cleaved-caspase-3 (Abcam, ab2302, 1:1000), RRM2 (Abcam, ab57653, 1:1000), and GAPDH (Abcam, ab181602, 1:3000). Goat anti-rabbit/mouse IgG H&L (HRP) secondary antibody (Abcam, ab205718/ab205719, 1:5000) acted as a secondary antibody.

### Wound healing assay

CaSki and SiHa cells were seeded into 6-well plates with 2 × 10^5^/well, and the monolayer cells were scratched to generate two straight wounds using a sterile 200 μL pipette tip. The scraped cells were removed by phosphate buffer solution (PBS; Sigma-Aldrich), and then, cells were incubated with a serum-free medium for 24 h. The wound widths at 0 h and 24 h were measured, followed by the calculation of wound healing rate: (width at 0 h – width at 24 h)/width at 0 h × 100%.

### Transwell assay

Migrated cells were detected by the transwell chamber (Corning Inc., Corning, NY, USA), and the transwell chamber was enveloped with matrigel (BD Biosciences) for invasion determination [23]. The upper chamber was added with 1 × 10^5^ CaSki and SiHa cells, and then, 500 μL cell medium was pipetted into the lower chamber. The transwell chamber was incubated for 24 h; then cells from the upper chamber into the lower chamber were stained with 0.1% crystal violet (Beyotime). Cell pictures were saved at 100× magnification by an inverted microscope (Olympus), and cell number was counted under three fields of view.

### RNA pull-down assay

C-1 magnetic beads (Life Technologies, Carlsbad, CA, USA) were conjugated with oligo probe or circDHTKD1 probe (RIBOBIO), followed by incubation with CaSki and SiHa cells at 4°C overnight. Then, miRNA levels were examined via RT-qPCR. For target binding between circ_0008460 and miR-197-3p, cells were transfected with biotin-coupled miR-197-3p mimic (Bio-miR-197-3p) or biotin-coupled mimic control (Bio-NC) and incubated with magnetic beads. The expression of circ_0008460 was measured via RT-qPCR.

### Dual-luciferase reporter assay

Starbase software (http://starbase.sysu.edu.cn) was exploited for the prediction of binding sites, and target interaction was analyzed using dual-luciferase reporter assay [24]. To construct luciferase plasmids, circ_0008460 and RRM2 3ʹUTR sequences were respectively inserted into pmirGLO (Promega, Madison, WI, USA). Wild-type (WT) plasmids with miR-197-3p binding sites were named as circ_0008460 WT and RRM2 3ʹUTR WT, while mutant-type (MUT) plasmids with mutated miR-197-3p binding sites were denoted circ_0008460 MUT and RRM2 3ʹUTR MUT. Circ_0008460 or RRM2 plasmids were co-transfected with miR-NC or miR-197-3p; then, 2 × 10^5^ CaSki and SiHa cells were harvested after transfection for 48 h. Luciferase activity detection was carried out by Dual-Luciferase Reporter Kit (Promega) following user’s manuals.

### Xenograft tumor assay

BALB/c nude mice (Vital River Laboratory Animal Technology Co., Ltd., Beijing, China) were divided into three groups (lenti-NC, OMT+lenti-NC, and OMT+lenti-circ_0008460), with 6 mice in each group. Mice were subcutaneously injected with 2 × 10^6^ SiHa cells of lenti-NC or lenti-circ_0008460 group and then treated with 150 mg/kg OMT by a gastric way. Tumor size was observed every 7 d, and volume was calculated by the following formula: Length × Width^2^/2. After 35 d, the flow rate of CO_2_ was used for euthanasia of mice and tumors were dissected from mice. Circ_0008460, miR-197-3p, and RRM2 levels in tumors were determined via RT-qPCR and western blot. Moreover, Ki67 (Abcam, ab15580) protein analysis was performed via Immunohistochemistry (IHC) assay [25]. All protocols were performed in accordance with the Animal Ethical Committee of Northwest Women and Children Hospital.

### Statistical analysis

All cell experiments were repeated three times with three paralleled samples. Data were represented as mean ± standard deviation and then data were analyzed through SPSS 22.0 (SPSS Inc., Chicago, IL, USA). Subsequently, Student’s *t*-test and analysis of variance (ANOVA) followed by Tukey’s test were exploited to analyze difference of groups. Statistically, *P* < 0.05 was indicated as a significant difference.

## Results

### OMT reduced circ_0008460 expression in CC cells

**Figure 1.**
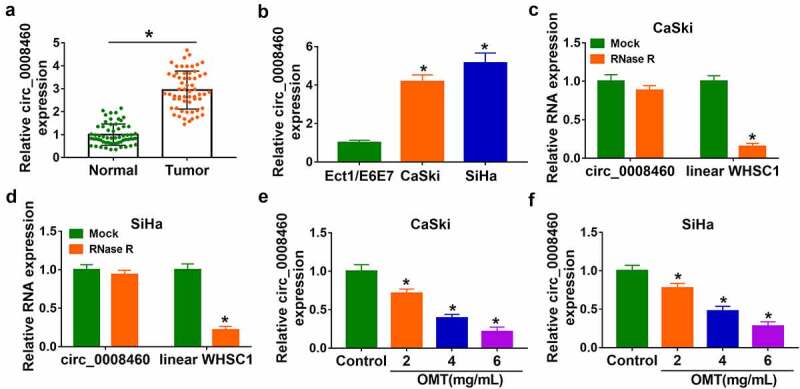
OMT reduced circ_0008460 expression in CC cells. (a-b) Circ_0008460 level was assayed via RT-qPCR in CC tissues (a) and CaSki/SiHa cells (b). (c-d) Linear WHSC1 and circ_0008460 levels were detected using RT-qPCR after RNase R treatment for total RNA. (e-f) Circ_0008460 expression was examined through RT-qPCR in control, 2 mg/mL, 4 mg/mL, and 6 mg/mL OMT groups of CaSki and SiHa cells. *P < 0.05.

OMT exhibited anti-tumor function in CC, and circRNAs can regulate gene levels via acting as miRNA sponges in CC progression. The purpose of this study was to investigate the association of OMT with circRNA/miRNA/mRNA axis. OMT was hypothesized to inhibit CC progression via targeting circ_0008460/miR-197-3p/RRM2 axis. Firstly, circ_0008460 quantification in CC was conducted using RT-qPCR. The data revealed that circ_0008460 expression was much higher in CC tissues than in normal controls (Figure 1(a)), as well as in CaSki and SiHa cells than in normal Ect1/E6E7 cells (Figure 1(b)). RNA stability was determined after total RNA was treated with RNase R. As shown in Figure 1(c,d), linear WHSC1 was significantly downregulated but circ_0008460 level was not affected by RNase R. Then, CaSki and SiHa cells were treated with different concentrations of OMT. The Circ_0008460 level was markedly inhibited in 2 mg/mL, 4 mg/mL, and 6 mg/mL OMT groups relative to the control group (Figure 1(e,f)). The 6 mg/mL group was used for OMT treatment in subsequent assays.

### Circ_0008460 abrogated OMT-induced inhibition of CC cell malignant behaviors

**Figure 2.**
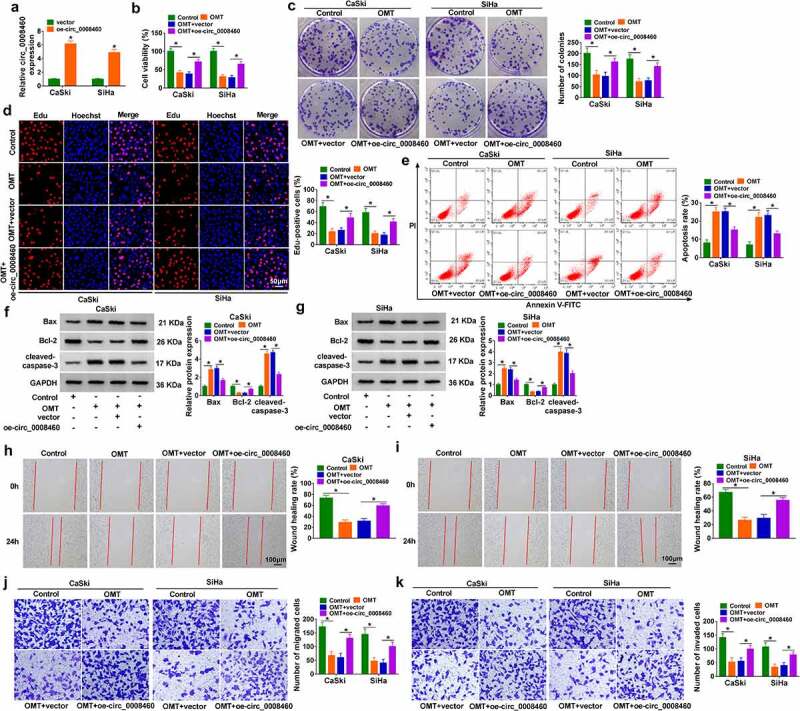
Circ_0008460 abrogated OMT-induced inhibition of CC cell malignant behaviors. (a) RT-qPCR was applied for assessing the overexpression efficiency of oe-circ_0008460. (b-k) CaSki and SiHa cells were divided into control, OMT (6 mg/mL), OMT+vector, and OMT+oe-circ_0008460 groups. (b) CCK-8 assay was used for determining cell viability. (c-d) Colony formation assay (c) and Edu assay (d) were employed for examining proliferation ability. (e) Flow cytometry was exploited for measuring the apoptosis rate. (f-g) Western blot was applied for assaying protein levels of apoptosis-related markers. (h-i) Wound healing assay was used for detecting cell migration. (j-k) Transwell assay was employed for evaluating cell migration (j) and invasion (k). *P < 0.05.

RT-qPCR showed that the circ_0008460 level was obviously increased in the oe-circ_0008460 transfection group compared with the vector transfection group in CaSki and SiHa cells (Figure 2(a)). Treatment with OMT resulted in inhibiting influences on cell viability (Figure 2(b)), colony number (Figure 2(c)), and Edu-positive cells (Figure 2(d)), while cell growth inhibition was then reversed by oe-circ_0008460. Flow cytometry demonstrated that the cell apoptosis rate was decreased in the OMT+oe-circ_0008460 group in contrast to the OMT+vector group (Figure 2(e)). Furthermore, apoptotic proteins were examined via western blot. Transfection of oe-circ_0008460 counteracted OMT-mediated protein upregulation of Bax/cleaved-caspase 3 and downregulation of Bcl-2 in CaSki and SiHa cells (figure 2(f,g)). The wound healing rate was reduced by OMT treatment, which was evidently mitigated after circ_0008460 was upregulated (Figure 2(h,i)). Also, the suppressive effects of OMT on migrated and invaded cells were alleviated following overexpression of circ_0008460 (Figure 2(j,k)). Overall, OMT exhibited anti-tumor effects via downregulating circ_0008460 in CC cells.

### Circ_0008460 served as a natural sponge for miR-197-3p

**Figure 3.**
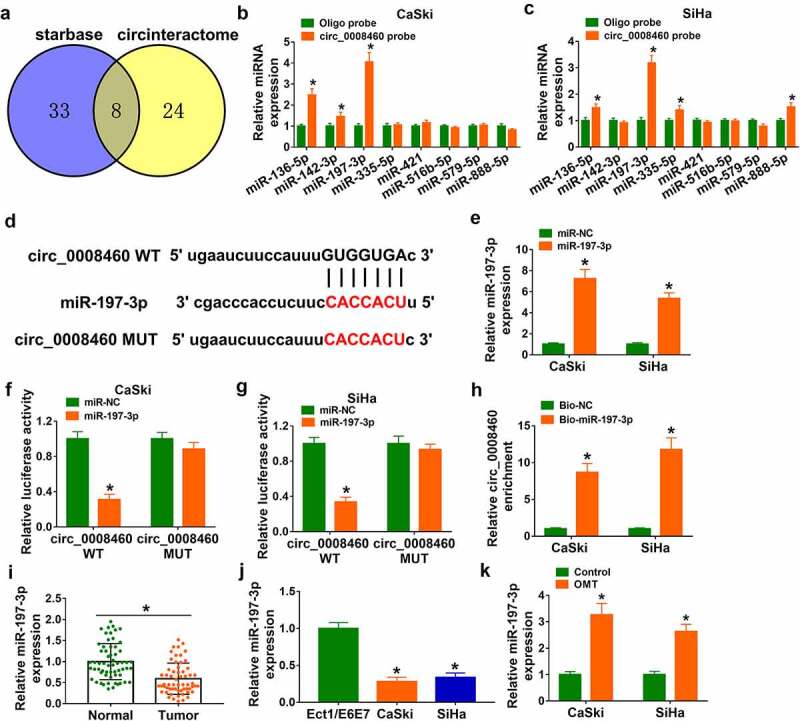
Circ_0008460 served as a natural sponge for miR-197-3p. (a) Venn Diagram analysis of miRNA targets from starbase and circinteractome. (b-c) The miRNA targets for circ_0008460 were screened by pull-down assay. (d) Circ_0008460 and miR-197-3p binding prediction in starbase. (e) The efficacy of miR-197-3p mimic was assessed via RT-qPCR. (f-h) Target binding between circ_0008460 and miR-197-3p was confirmed through dual-luciferase reporter assay (f-g) and RNA pull-down assay (h). (i-k) RT-qPCR was performed for miR-197-3p level quantification in CC tissues (i), CaSki/SiHa cells (j), and 6 mg/mL OMT-treated CC cells (k). NC: normal control, *P < 0.05.

Venn Diagram analysis indicated that eight miRNAs (miR-136-5p, miR-142-3p, miR-197-3p, miR-335-5p, miR-421, miR516b-5p, miR-579-5p, and miR-888-5p) were mutually predicted as potential targets of circ_0008460 from starbase and circinteractome software packages (Figure 3(a)). The RNA pull-down assay indicated that miR-136-5p and miR-197-3p were captured by circ_0008460 probe in both CaSki and SiHa cells (Figure 3(b,c)). MiR-197-3p with a more significant change (than miR-136-5p) was used for further target research. The binding region between circ_0008460 and miR-197-3p is shown in Figure 3(d). Overexpression of miR-197-3p was achieved by transfection of miR-197-3p, and the transfection efficiency was conspicuous in CaSki and SiHa cells (Figure 3(e)). Dual-luciferase reporter assay manifested that luciferase activity was repressed after miR-197-3p co-transfection with circ_0008460 WT, but there was no significant difference after co-transfection with miR-197-3p and circ_0008460 MUT (figure 3(f,g)). In addition, circ_0008460 was largely pulled down by Bio-miR-197-3p compared to the Bio-NC group (Figure 3(h)). Downregulation of miR-197-3p was detected in CC samples (Figure 3(i)) and CaSki/SiHa cells (Figure 3(j)), relative to normal control tissues and Ect1/E6E7 cells. The expression of miR-197-3p was upregulated after CaSki and SiHa cells were treated with 6 mg/mL OMT (Figure 3(k)). These evidences affirmed that miR-197-3p was a miRNA target of circ_0008460.

### OMT inhibited CC cell progression by regulating circ_0008460/miR-197-3p axis

**Figure 4.**
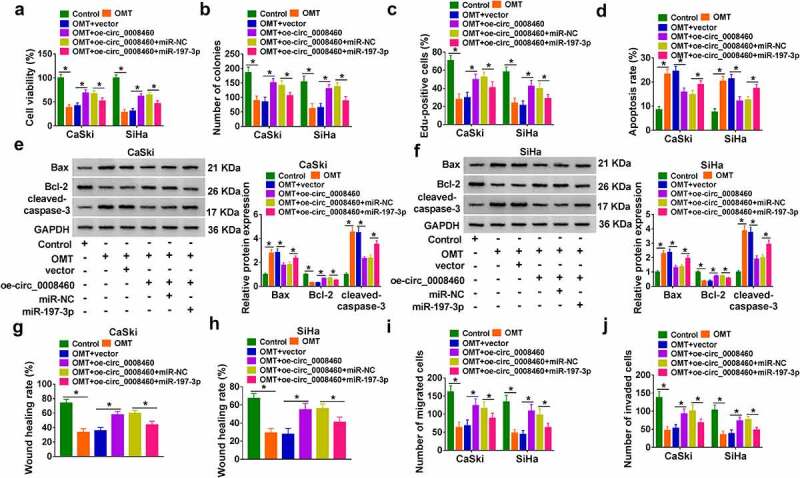
OMT inhibited CC cell progression by regulating circ_0008460/miR-197-3p axis. CaSki and SiHa cells were treated with control, OMT (6 mg/mL), OMT+vector, OMT+oe-circ_0008460, OMT+oe-circ_0008460+ miR-NC, and OMT+oe-circ_0008460 + miR-197-3p. (a) Cell viability was measured using CCK-8 assay. (b-c) Cell proliferation was evaluated via colony formation assay (b) and Edu assay (c). (d-f) Cell apoptosis was analyzed through flow cytometry (d) and western blot (e-f). (g-j) Cell motility was assessed by wound healing assay (g-h) and transwell assay (i-j). NC: normal control, *P < 0.05.

Furthermore, cell experiments were performed to explore whether circ_0008460 and miR-197-3p interaction was related to OMT in CC development. The reversal regulation of oe-circ_0008460 for OMT was attenuated by miR-197-3p mimic in cell viability (Figure 4(a)) and proliferation (Figure 4(b,c)). The results of flow cytometry (Figure 4(d)) and western blot (Figure 4(e,f)) suggested that miR-197-3p transfection abolished oe-circ_0008460-mediated apoptosis inhibition in OMT-treated cells. After analysis of wound healing assay (Figure 4(g,h)) and transwell assay (Figure 4(i,j)), we found that circ_0008460 offset OMT-induced suppression of cell motility via reducing miR-197-3p. Altogether, circ_0008460/miR-197-3p axis was involved in biological regulation of OMT in CC cells.

### Circ_0008460 regulated RRM2 expression via sponging miR-197-3p

**Figure 5.**
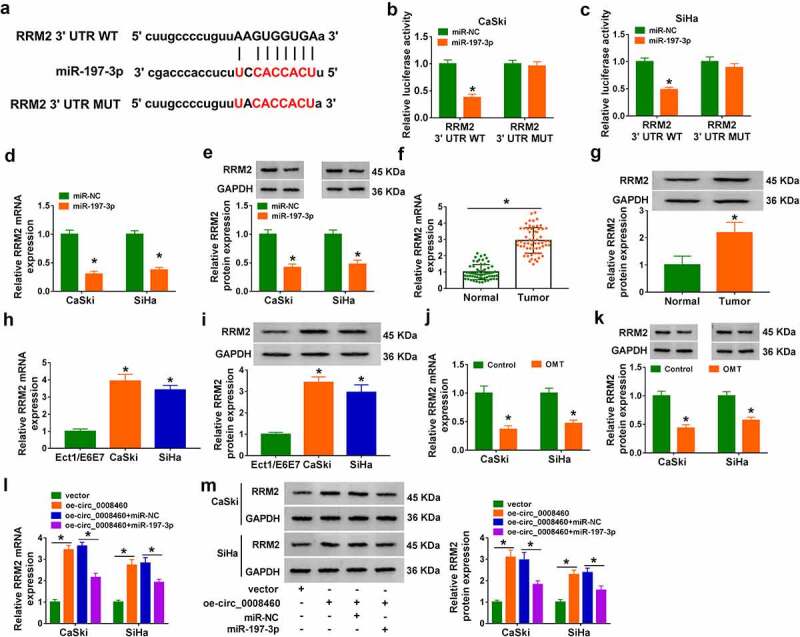
Circ_0008460 regulated RRM2 expression via sponging miR-197-3p. (a) RRM2 3ʹUTR sequence was predicted to have binding sites with miR-197-3p sequence in starbase. (b-c) Dual-luciferase reporter assay was used for interaction analysis between miR-197-3p and RRM2 3ʹUTR in CaSki and SiHa cells. (d-e) RT-qPCR and western blot were conducted for level examination of RRM2 after miR-NC or miR-197-3p transfection. (f-k) RRM2 mRNA and protein levels were determined in CC samples (f-g), CaSki/SiHa cells (h-i), and OMT-exposed CC cells (j-k) using RT-qPCR and western blot. (l-m) RRM2 mRNA and protein detection was performed via RT-qPCR and western blot in vector, oe-circ_0008460, oe-circ_0008460 + mi-NC, and oe-circ_0008460+ miR-197-3p groups. NC: normal control, *P < 0.05.

Starbase predicted the binding sites between miR-197-3p and RRM2 3ʹUTR sequences (Figure 5(a)). Overexpression of miR-197-3p evoked an inhibitory effect on the luciferase activity of the RRM2 3ʹUTR WT plasmid, but it did not affect the luciferase activity of the RRM2 3ʹUTR MUT plasmid (Figure 5(b,c)). RRM2 mRNA and protein levels were overtly inhibited in miR-197-3p-transfected CaSki and SiHa cells relative to miR-NC-transfected cells (Figure 5(d,e)). RT-qPCR and western blot data confirmed that RRM2 was highly expressed in CC tissues (figure 5(f,g)) and CaSki/SiHa cells (Figure 5(h,i)), compared with normal tissues and Ect1/E6E7 cells. In addition, OMT treatment triggered mRNA and protein downregulation of RRM2 in CaSki and SiHa cells (Figure 5(j,k)). High level of circ_0008460 upregulated mRNA and protein levels of RRM2, whereas miR-197-3p transfection abated this regulation (Figure 5(l,m)). The clinical data indicated that circ_0008460 and RRM2 were associated with tumor growth and metastasis, while miR-197-3p exhibited anti-tumor function in CC patients (Tables 2,3 and 4). Hence, circ_0008460 sponged miR-197-3p to promote RRM2 expression in CC progression.

**Table 2.** Correlation between clinicopathologic parameters of cervical cancer patients and circ_0008460 expression

		Circ_0008460 expression		*P* value
Parameter	Case	Low (*n* = 30)	High (*n* = 30)	
Age (years)				0.297
≤45	26	11	15	
>45	34	19	15	
Tumor size				0.038*
≤4 cm	26	20	12	
>4 cm	21	10	18	
Differentiation				0.069
Good/moderate	21	17	10	
Poor	26	13	20	
Lymph-node metastasis				0.0002*
Yes	22	18	4	
No	38	12	26	

*P < 0.05.

**Table 3.** Correlation between clinicopathologic parameters of cervical cancer patients and miR-197-3p expression

		miR-197-3p expression		*P* value
Parameter	Case	Low (*n* = 30)	High (*n* = 30)	
Age (years)				0.602
≤45	34	16	18	
>45	26	14	12	
Tumor size				0.004*
≤4 cm	27	19	8	
>4 cm	33	11	22	
Differentiation				0.793
Good/moderate	25	12	13	
Poor	35	18	17	
Lymph-node metastasis				0.0003*
Yes	21	20	6	
No	26	10	24	

*P < 0.05.

**Table 4.** Correlation between clinicopathologic parameters of cervical cancer patients and RRM2 expression

		RRM2 expression		*P* value
Parameter	Case	Low (*n* = 30)	High (*n* = 30)	
Age (years)				0.292
≤45	36	20	16	
>45	24	10	14	
Tumor size				0.018*
≤4 cm	35	22	13	
>4 cm	25	8	17	
Differentiation				0.284
Good/moderate	22	13	9	
Poor	38	17	21	
Lymph-node metastasis				0.0004*
Yes	27	19	8	
No	33	11	22	

*P < 0.05.

### OMT exhibited anti-cancer function in CC cells by upregulating miR-197-3p to downregulate RRM2

**Figure 6.**
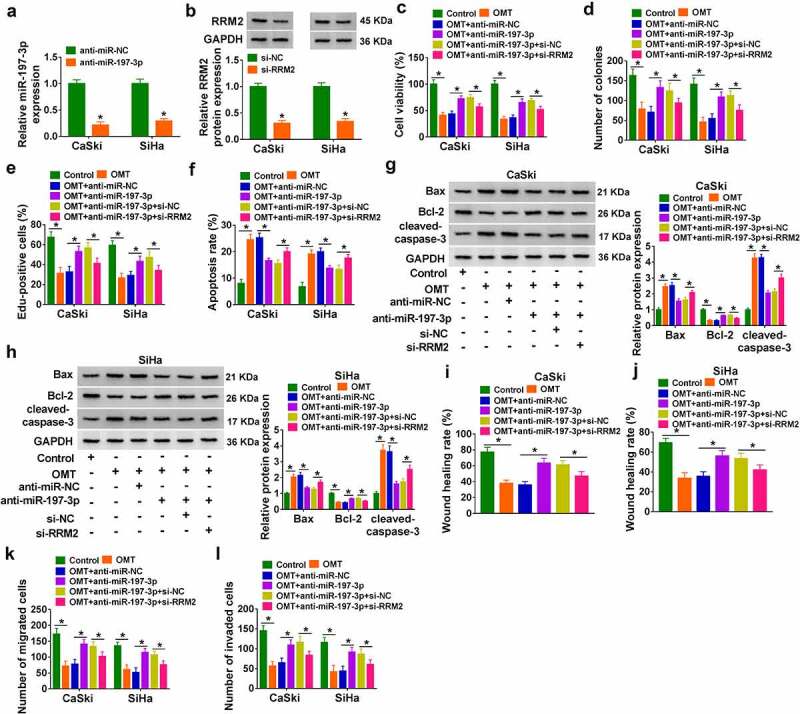
OMT exhibited anti-cancer function in CC cells by upregulating miR-197-3p to downregulate RRM2. (a-b) Transfection efficiencies of anti-miR-197-3p (a) and si-RRM2 (b) were examined by RT-qPCR and western blot, respectively. (c-l) 6 mg/mL-treated CaSki and SiHa cells were transfected with anti-miR-197-3p, anti-miR-197-3p+si-RRM2, or relative controls. (c) CCK-8 assay was performed for viability detection. (d-e) The examination of cell proliferation was conducted using colony formation assay (d) and Edu assay (e). (f-h) Cell apoptosis analysis was implemented through flow cytometry (f) and western blot (g-h). (i-l) Wound healing assay (i-j) and transwell assay (k-l) were performed to assess cell motility. NC: normal control, *P < 0.05.

RT-qPCR and western blot analysis demonstrated that the inhibitory efficiencies of anti-miR-197-3p (Figure 6(a)) and si-RRM2 (Figure 6(b)) were excellent in CaSki and SiHa cells. OMT-induced cell viability reduction (Figure 6(c)) and proliferation inhibition (Figure 6(d,e)) were attenuated by anti-miR-197-3p transfection, while si-RRM2 further counterbalanced the regulation of anti-miR-197-3p in cell growth. Inhibition of miR-197-3p reduced the apoptosis rate (figure 6(f)) and reversed expression changes of apoptotic markers (Figure 6(g,h)) in OMT-treated cells; then, these influences were eliminated after knockdown of RRM2. OMT-induced suppression of the wound healing rate (Figure 6(i,j)) and reduction of migrated or invaded cells (Figure 6(k,l)) were also lightened by anti-miR-197-3p, which was subsequently abrogated by the silence of RRM2 expression. These findings demonstrated that OMT function was partly ascribed to miR-197-3p/RRM2 axis.

### *OMT inhibited tumor growth of CC by downregulating circ_0008460 to mediate miR-197-3p/RRM2 axis* in vivo

**Figure 7.**
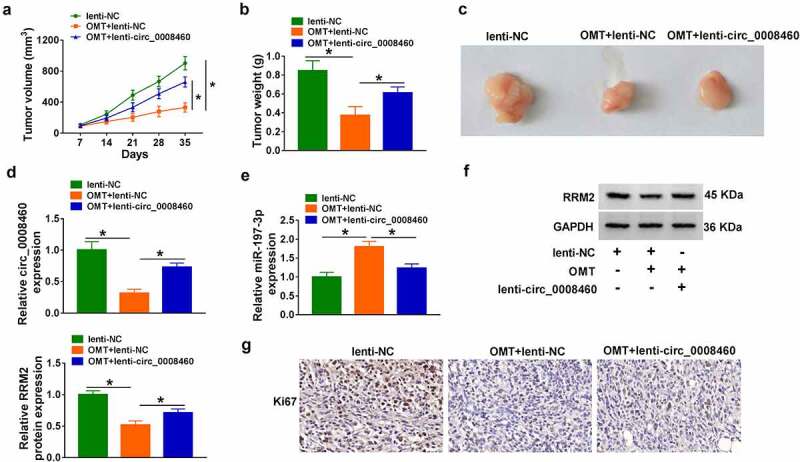
OMT inhibited tumor growth of CC by downregulating circ_0008460 to mediate miR-197-3p/RRM2 axis *in vivo*. CaSki xenograft models of lenti-NC, OMT+lenti-NC, and OMT+lenti-circ_0008460 were constructed in mice. (a-b) Tumor volume (a) and weight (b) in nude mice. (c) Images of tumor tissues. (d-e) Circ_0008460 (d) and miR-197-3p (e) levels were assayed through RT-qPCR. (f-g) Western blot and IHC assays were used for protein determination of RRM2 (f) and Ki67 (g) in tumors. NC: normal control, *P < 0.05.

Tumor volume (Figure 7(a)) and weight (Figure 7(b)) showed that tumor growth was reduced by OMT treatment, while this effect was alleviated by lenti-circ_0008460 in mice. Tumor images are displayed in Figure 7(c). OMT-induced circ_0008460 downregulation (Figure 7(c)), miR-197-3p upregulation (Figure 7(e)), and RRM2 protein inhibition (figure 7(f)) were all recovered by lenti-circ_0008460 in tumor tissues. Moreover, the IHC assay demonstrated that the inhibition of Ki67 positive rate by OMT was attenuated in the OMT+lenti-circ_0008460 group (Figure 7(g)). Taken together, OMT resulted in tumor growth repression by targeting circ_0008460/miR-197-3p/RRM2 axis *in vivo*.

## Discussion

Herein, circ_0008460 was elucidated to regulate the anti-tumor function of OMT in CC progression by targeting miR-197-3p/RRM2 axis. The regulatory network for OMT in CC was disclosed, and circ_0008460/miR-197-3p/RRM2 axis might improve the treatment of OMT for CC.

OMT exhibits various therapeutic regulations in human diseases, including anti-cancer, anti-viral, and anti-inflammatory effects [26]. For instance, OMT effectively suppressed epithelial-mesenchymal transition and proliferation of breast cancer cells [27,28]. Ni *et al*. asserted that OMT induced cell death and reduced cell growth in nasopharyngeal cancer [29]. Liang *et al*. found that migration and proliferation capacities of colorectal cancer cells were blocked by OMT [30]. In addition, anti-tumor effects of OMT were identified in lung cancer and ovarian cancer [31,32]. Our biological assays demonstrated that OMT restrained CC cell viability, proliferation, and motility. The apoptosis rate and apoptotic protein levels showed that OMT induced CC cell apoptosis. These evidences affirmed the anti-cancer role of OMT in CC progression, which was consistent with the previous findings [4,33].

CircRNAs have acted as key biomolecules in CC progression. CircZFR was shown to accelerate cell cycle progression and cell proliferation in CC [34]. Circ_0003204 overexpression resulted in CC cell growth and invasion promotion [35]. Differently, circSmarca5 and circ_0043280 served as cancer inhibitors for CC development [36,37]. In this study, we detected the significant upregulation of circ_0008460 in CC. Interestingly, the circ_0008460 level was reduced by OMT in CC cells. OMT-induced anti-cancer effects on CC cells were all reversed after circ_0008460 knockdown, suggesting that circ_0008460 played an oncogenic role in CC and OMT-inhibited CC progression was partly achieved by downregulating circ_0008460. Through reducing the level of circ_0008460, the inhibitory function of OMT in CC can be enhanced. In addition, circ_0008460 might be used as a prognostic marker after OMT treatment.

CircRNAs have important interaction with miRNAs in cancer regulation. Sun *et al*. stated that circ_0082835 accelerated malignant progression and lymphatic metastasis via serving as a miR-429 sponge in primary melanoma [38]. Wang *et al*. declared that circRNA-000911 retarded invasion and enhanced apoptosis through inhibiting miR-449a expression in breast cancer cells [39]. In CC, circ-Smarca5 and circ_0000263 acted as sponges of miR-432 and miR-150-5p, respectively [40,41]. Currently, miR-197-3p was verified to be a miRNA target for circ_0008460. Furthermore, the reversal regulation of circ_0008460 for OMT function was attenuated by miR-197-3p. OMT inhibited CC cell progression by targeting circ_0008460/miR-197-3p axis.

OMT was found to promote apoptosis and repress proliferation in ovarian cancer via upregulating miR-29b to reduce the level of MMP-2 [31]. Additionally, OMT restrained gastric cancer cell progression via mediating miR-93-5p/AHNAK axis [42]. RRM2 has been identified as an oncogene in CC and associated with anti-tumor roles of miRNAs, such as miR-140-3p and miR-5095 [16,17]. Also, we confirmed that miR-197-3p/RRM2 axis was associated with OMT-induced cancer inhibition of CC cells. CircRNAs can regulate gene expression by sponging miRNAs in human cancers. For example, circMMD_007 promoted the development of lung adenocarcinoma by targeting miR-197-3p to increase protein tyrosine phosphatase non-receptor type 9 (PTPN9) expression [43]. CircZNF609 upregulated E2F6 via interacting with miR-197-3p to function as an oncogene in CC [13]. Moreover, circ_0008460 triggered significant upregulation of RRM2 via targeting miR-197-3p. *In vivo* assay further manifested that OMT reduced tumor growth by resulting in downregulation of circ_0008460 to regulate miR-197-3p and RRM2 levels. It is potential to improve the therapeutic effect of OMT via targeting circ_0008460 to mediate miR-197-3p/RRM2 axis.

However, this study still has some limitations. Firstly, the different signaling pathways in the downstream of circ_0008460/miR-197-3p/RRM2 axis remain to be researched. Secondly, there may be other miRNA/mRNA axes for circ_0008460 and it will be explored in the future study.

## Conclusion

In conclusion, OMT induced the downregulation of circ_0008460 to upregulate the level of miR-197-3p and reduce the expression of RRM2 to regulate the malignant behaviors of CC cells including proliferation, apoptosis, and migration or invasion (Graphical abstract). For the first time, the regulatory function of OMT in CC progression was confirmed to be partly associated with circ_0008460/miR-197-3p/RRM2 signal axis. This study elucidated the molecular pathway underlying the anti-tumor effect of OMT, which might provide the further understanding of the functional mechanism of OMT in the malignant development of CC. Moreover, circ_0008460/miR-197-3p/RRM2 axis might be used to improve the treatment of OMT.

## Supplementary Material

Supplemental Material
