# Supplementary figures and images for: Oxymatrine induces anti-tumor response in cervical cancer by modulating circ_0008460/miR-197-3p/ribonucleotide reductase subunit M2 (RRM2)
Source: Bioengineered. 2022 May 24;13(5):12912–26. doi: 10.1080/21655979.2022.2078943 (PMC9275878; doi:10.1080/21655979.2022.2078943)

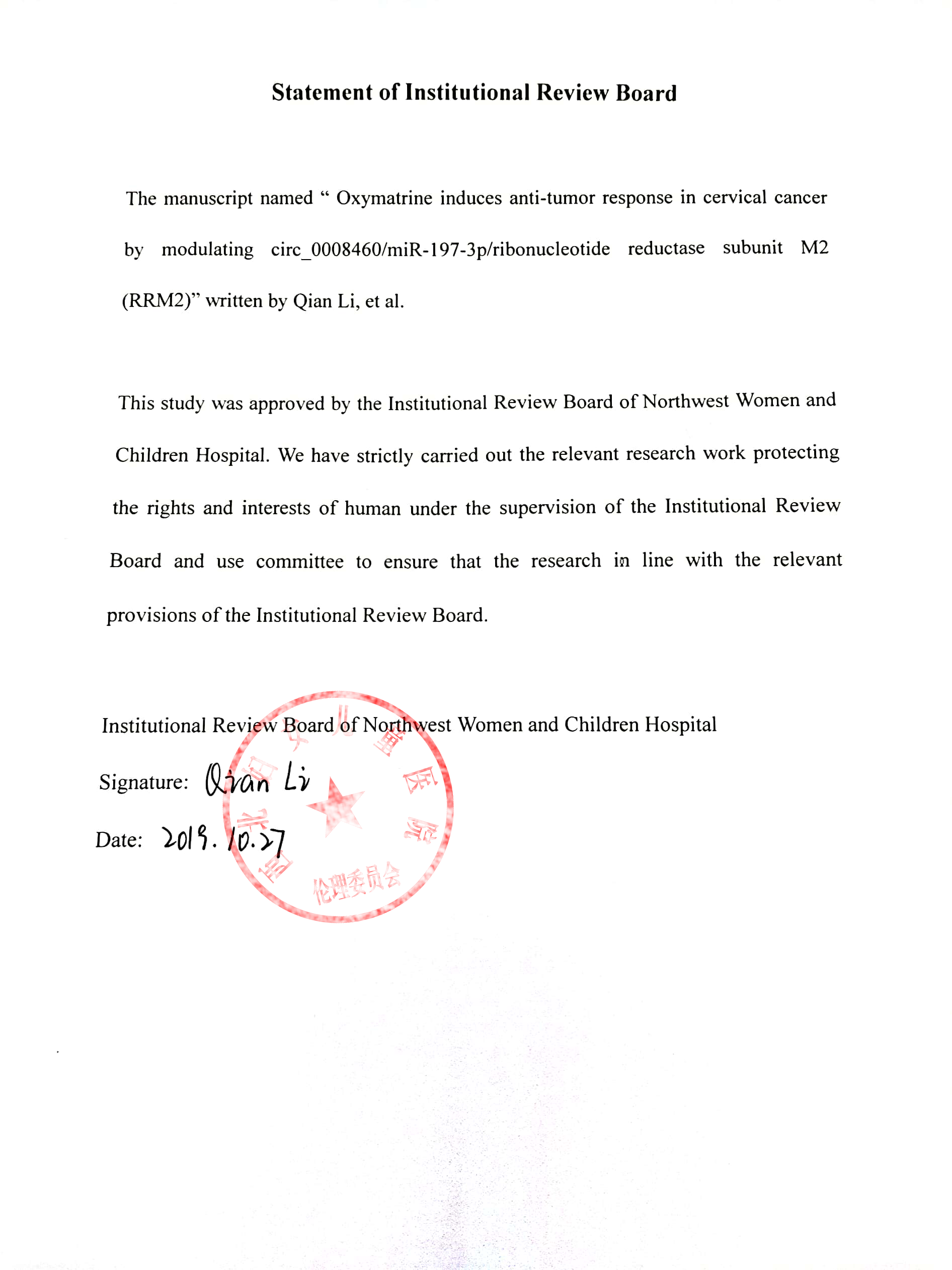

Supplement: Supplemental Material [file KBIE_A_2078943_SM3662.zip › supplementary/IRB.docx]
